# Effects of radiotherapy after hyperbaric oxygenation on malignant gliomas

**DOI:** 10.1038/sj.bjc.6690345

**Published:** 1999-04

**Authors:** K Kohshi, Y Kinoshita, H Imada, N Kunugita, H Abe, H Terashima, N Tokui, S Uemura

**Affiliations:** Departments of Neurosurgery, andRadiologySecond Department of Internal Medicine and Division of Hyperbaric Medicine, Department of Clinical Epidemiology, Institute of Industrial Ecological Sciences, University of Occupational and Environmental Health, 1-1 Iseigaoka, Yahatanishi-ku, Kitakyushu 807-8555, Japan; Department of Neurosurgery, Amakusa Medical Center, Hondo, Japan

**Keywords:** malignant glioma, hyperbaric oxygenation, radiation therapy, clinical trials

## Abstract

The purpose of this non-randomized trial was to evaluate the efficacy of radiotherapy combined with hyperbaric oxygen (HBO) in patients with malignant glioma. Between 1987 and 1997, 29 patients in whom computerized tomography (CT) or magnetic resonance imaging (MRI) scans showed post-operative residual tumours were locally irradiated with nitrosourea-based chemotherapy. Treatments were consecutively combined with HBO at two institutions since 1991 and 1993. Fifteen patients were irradiated daily after HBO, and the periods of time from decompression to irradiation were within 15 and 30 min in 11 and four patients respectively. Fourteen other patients were treated without HBO. Tumour responses were assessed by CT or MRI scans and survival times were compared between the treated groups. In the HBO group, 11 of 15 patients (73%) showed ≥ 50% tumour regression. All responders were irradiated within 15 min after decompression. In the non-HBO group, four of 14 patients (29%) showed tumour regression. The median survivals in patients with and without HBO were 24 and 12 months, respectively, and were significantly different (*P* < 0.05). No serious side-effects were observed in the HBO patients. In conclusion, irradiation after HBO seems to be a useful form of treatment for malignant gliomas, but irradiation should be administered immediately after decompression. © 1999 Cancer Research Campaign


British Journal of Cancer (1999) 80(1/2), 236–241
© 1999 Cancer Research Campaign
Article no. bjoc.1998.0345

### Effects of radiotherapy after hyperbaric oxygenation on malignant gliomas

**K Kohshi[1,4], Y Kinoshita[1], H Imada[2], N Kunugita[2], H Abe[3], H Terashima[2], N Tokui[5] and S Uemura[6]**

Departments of [1]Neurosurgery and [2]Radiology, [3]Second Department of Internal Medicine and [4]Division of Hyperbaric Medicine, [5]Department of Clinical Epidemiology, Institute of Industrial Ecological Sciences, University of Occupational and Environmental Health, 1-1 Iseigaoka, Yahatanishi-ku, Kitakyushu 807-8555, Japan; [6]Department of Neurosurgery, Amakusa Medical Center, Hondo, Japan

**Summary** The purpose of this non-randomized trial was to evaluate the efficacy of radiotherapy combined with hyperbaric oxygen (HBO) in patients with malignant glioma. Between 1987 and 1997, 29 patients in whom computerized tomography (CT) or magnetic resonance imaging (MRI) scans showed post-operative residual tumours were locally irradiated with nitrosourea-based chemotherapy. Treatments were consecutively combined with HBO at two institutions since 1991 and 1993. Fifteen patients were irradiated daily after HBO, and the periods of time from decompression to irradiation were within 15 and 30 min in 11 and four patients respectively. Fourteen other patients were treated without HBO. Tumour responses were assessed by CT or MRI scans and survival times were compared between the treated groups. In the HBO group, 11 of 15 patients (73%) showed ≥ 50% tumour regression. All responders were irradiated within 15 min after decompression. In the non-HBO group, four of 14 patients (29%) showed tumour regression. The median survivals in patients with and without HBO were 24 and 12 months, respectively, and were significantly different ($P < 0.05$). No serious side-effects were observed in the HBO patients. In conclusion, irradiation after HBO seems to be a useful form of treatment for malignant gliomas, but irradiation should be administered immediately after decompression.

**Keywords:** malignant glioma; hyperbaric oxygenation; radiation therapy; clinical trials

Malignant gliomas, comprising the majority of primary brain tumours in adults, have a very poor prognosis. Traditional therapies, such as surgery, radiotherapy and some chemotherapeutic agents, have been used in various combinations in the search for a curative regimen. Radiotherapy has a major role in the adjuvant therapies for malignant gliomas. Despite notable technical advances in both the surgical and radiotherapeutic treatment approaches to malignant gliomas during the last decade, patient survival has not improved. The presence of hypoxic tumour cells is thought to be a major reason for tumour resistance to radiotherapy (Gray et al, 1953; Hall, 1994).

Molecular oxygen has long been recognized as one of the most powerful modifiers of cellular radiation sensitivity. For example, oxygen has been reported to increase, by a factor of approximately 3, the biological effect of ionizing radiation on mammalian cells in which radiation is performed under well-oxygenated conditions as compared to anoxic conditions (Gray et al, 1953). Thus hyperbaric oxygen (HBO) exposure, which improves the oxygen supply to hypoxic tumour cells, was used in combination with radiotherapy to treat malignant gliomas (Chang, 1977; Dowling et al, 1992). The previous combination method in which irradiation was administered during HBO exposure was hazardous to patients and a complex technique, and as a result HBO has not been routinely adopted with radiotherapy to treat malignant gliomas (Chang, 1977; Jain, 1990; Dische, 1991).

*Received 24 February 1998*
*Revised 7 August 1998*
*Accepted 22 October 1998*

*Correspondence to:* K Kohshi

Exposure to HBO produces tissue oxygenation, and the increased $P\mathrm{O}_2$ persists in normal tissues for certain periods after decompression (Wells et al, 1977). We hypothesized that if $P\mathrm{O}_2$ in malignant gliomas remains elevated after HBO exposure, the resulting improvement in irradiation after decompression may lead to enhanced tumour control. In our previous pilot study, we reported that all patients who received HBO experienced regression of their residual tumours (Kohshi et al, 1996). Consequently, we evaluated the effectiveness and safety of this new approach to treat malignant gliomas.

### MATERIALS AND METHODS

### Patient selection

At our University Hospital of Occupational and Environmental Health between 1987 and 1997, and at Amakusa Medical Center between 1990 and 1997, 72 cases with newly diagnosed malignant gliomas were locally irradiated after tumour debulking or biopsy. Patients have been consecutively treated by radiotherapy combined with HBO at the above two institutions since 1991 and 1993 respectively. Eligibility criteria included:

1. all patients were above the age of 16 years
2. anaplastic astrocytoma or glioblastoma multiforme was pathologically verified according to the World Health Organization classification (Kleihues et al, 1993)
3. measurable disease was exhibited on pre-treatment contrast-enhanced computerized tomography (CT) or magnetic resonance imaging (MRI) scan
4. Karnofsky Performance Scale (KPS) score was 60 or greater at the time of assignment (Karnofsky et al, 1948)

5. patients treated with HBO had no cardiopulmonary diseases or sinusitis.

Patients were excluded if they had no residual tumour. Eligible patients were not to have received any previous radiotherapy or chemotherapy, but conventional doses of corticosteroids and anticonvulsants were allowed. Informed consent was obtained from all patients.

### Protocol

Radiotherapy began within 2 weeks after surgery in conjunction with chemotherapy as prescribed below. The irradiated field was determined by defining the volume of the tumour on CT or MRI scans prior to initiation of radiotherapy and allowing for a margin of at least 2 cm, but sparing as much brain as possible. External-beam radiotherapy was performed, using 10 MV X-rays from linear accelerators with a total dose of 50–71 Gy in 20–30 fractions, for 5 days per week over 5–6 weeks. To determine the effectiveness of the dose on the tumour, the biologically effective dose (BED) was calculated according to the guidelines of Fowler (Fowler, 1989) and were expressed in units of 10 Gy, using an $\alpha$–$\beta$ of 10, according to the formula: BED = total dose $\times$ (1 + fraction dose/$\alpha$–$\beta$). Exposure to HBO was administered in hyperbaric chambers according to the following schedule: 15 min of compression with air, 60 min of 100% oxygen inhalation using an oxygen mask at 2.5 atmospheres absolute and 10 min of decompression with oxygen inhalation. Each irradiation was administered daily after HBO exposure. The periods of time from decompression to irradiation were different in the two institutions, and were within 15 and 30 min after decompression. Corticosteroids were used perioperatively, during an early phase of radiotherapy, and as necessary thereafter. Supportive chemotherapy was performed in all patients and was similar for the two treatment groups. All patients were given nitrosourea as nimustine or ranimustine intravenously at a dose of 75 mg $m^{-2}$. The first dose was scheduled for the beginning of the radiotherapy and the second dose was administered 5 or 6 weeks later. Some patients in each treatment group received administration of interferon-$\beta$.

### Evaluation of response and statistical analysis

Clinical and neuroimaging follow-up evaluations were obtained for all patients 2–76 months after the completion of radiotherapy. CT and/or MRI scans were performed every 3 months for 1 year after radiotherapy, and then at 6-month intervals thereafter as indicated. The neurological status and the occurrence of complications were monitored in all patients. We estimated the amount of mass reduction by comparing contrast-enhanced CT or MRI scans obtained immediately after surgery with those obtained periodically after the completion of radiotherapy. The tumour size was defined for this study as the area of enhancing mass lesion, and was measured as the lesion size in the product of perpendicular diameters of each slice. The product of these values was calculated to provide an approximation of tumour area. Although this value is not an accurate measure of absolute tumour volume, it is adequate for use in comparing relative size from different scans from the same patient. Complete response was defined as the disappearance of all visible tumour. Partial response was defined as at least 50% regression of the tumour. No response was considered as less than 50% regression or progression of the contrast-enhanced mass. Complete and partial responses were defined as indications of effective treatment, and the ‘response rate’ was calculated as the percentage of cases with complete and partial responses. Tumour recurrence was defined as progression after partial or complete response.

We investigated the differences in patient characteristics and treatment parameters between the treated groups with and without HBO using the Student’s *t*-test or $\chi^2$ test. Survival curves were calculated using the Kaplan–Meier method (Kaplan and Meier, 1958). Differences between survival curves were determined using the log-rank test (Mantel, 1966). The Cox proportional hazards model was used to calculate the relative risk of each variable for survival (Cox, 1972). Results were considered significant for *P*-values < 0.05 and data were analysed using the SAS computer software package (SAS Institute, Cary, NC, USA).

**Table 1** Clinical characteristics of 29 patients with newly diagnosed malignant glioma

| Variables | Treatment group: With HBO (*n* = 15) | Treatment group: Without HBO (*n* = 14) | Significance |
|---|---|---|---|
| Age at diagnosis (years) | | | |
| Mean ± s.d. | 55.5 ± 19.0 | 57.9 ± 6.8 | NS |
| Gender | | | |
| Male/female | 8/7 | 9/5 | NS |
| KPS score | | | |
| 60–70 | 8 | 4 | NS |
| 80–100 | 7 | 10 | |
| Mental status | | | |
| Normal/abnormal | 7/8 | 8/6 | NS |
| Diagnosis procedure | | | |
| Partial removal | 9 | 13 | 0.082 |
| Biopsy only | 6 | 1 | |
| Histological category | | | |
| Anaplastic astrocytoma | 5 | 3 | NS |
| Glioblastoma multiforme | 10 | 11 | |
| Tumour location | | | |
| Diencephalon | 12 | 13 | |
| Thalamus | 2 | 0 | NS |
| Cerebellum | 1 | 1 | |
| Radiation from symptom (months) | | | |
| Mean ± s.d. | 2.7 ± 1.6 | 2.8 ± 1.4 | NS |
| Radiation dose (Gy) | | | |
| Mean ± s.d. | 57.8 ± 5.7 | 58.7 ±3.7 | NS |
| BED | | | |
| Mean ± s.d. | 71.5 ± 7.9 | 71.8 ± 3.9 | NS |
| Other treatments | | | |
| NU | 12 | 10 | NS |
| NU+FN | 3 | 4 | |

HBO: hyperbaric oxygenation, KPS: Karnofsky Performance Scale, BED: biologically effective dose, NS: not significant, NU: nitrosourea, IFN: interferon-$\beta$, *P*-values were determined using $\chi^2$ test.

### RESULTS

### Patient characteristics

Twenty-nine patients were enrolled in the study (23 patients from University Hospital of Occupational and Environmental Health

© Cancer Research Campaign 1999

**Table 2** Radiographic best response to treatment in 29 patients with newly diagnosed malignant glioma

| Treatment group | Response CR | PR | NR | Response rate[c] (%) | Comparison of[d] response rate |
|---|---|---|---|---|---|
| With HBO | 3[a] | 8[a] | 4[b] | 73 | |
| ($n$ = 15) | | | | | $P$ < 0.05 |
| Without HBO | 1 | 3 | 10 | 29 | |
| ($n$ = 14) | | | | | |

HBO: hyperbaric oxygenation; CR: complete response, PR: partial response, NR: no response; [a]Irradiated within 15 min after HBO, [b]irradiated 30 min after HBO, [c]response rates were calculated as the percentage of cases with complete and partial responses, [d]response rates were compared using $\chi^2$ test.

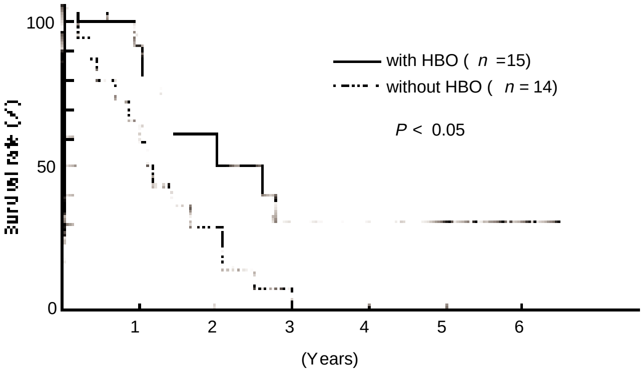

**Figure 1** Kaplan–Meier survival curve according to different schedules of treatment: with or without HBO (hyperbaric oxygenation). The curves are significantly different at $P$ < 0.05

**Table 3** Univariate analysis of prognostic variables for the survival of patients

| Variables | Univariate RR | (95% CI) | $P$-value |
|---|---|---|---|
| Age | 1.046 | 1.004–1.089 | 0.0310 |
| Gender | 1.599 | 0.659–3.877 | 0.2995 |
| KPS score | 0.983 | 0.946–1.021 | 0.3768 |
| Mental status | 1.856 | 0.778–4.429 | 0.1636 |
| Diagnosis procedure | 0.935 | 0.274–3.195 | 0.9350 |
| Histological category | 5.581 | 1.584–19.660 | 0.0074 |
| Tumour location | 0.149 | 0.020–1.126 | 0.0651 |
| Radiation from symptom | 1.663 | 0.666–4.152 | 0.2758 |
| Radiation dose | 1.677 | 0.643–4.377 | 0.2907 |
| BED | 0.653 | 0.261–1.631 | 0.3614 |
| Chemotherapeutic agents | 0.709 | 0.252–1.993 | 0.5141 |
| Treatment group | 0.348 | 0.137–0.883 | 0.0264 |

RR: relative risk, 95% CI: 95% confidence interval, KPS: Karnofsky performance scale, BED: biologically effective dose.

**Table 4** Multivariate analysis of prognostic variables for the survival of patients

| Variables | Multivariate RR | (95% CI) | $P$-value |
|---|---|---|---|
| Age | 1.050 | 0.985–1.119 | 0.1355 |
| Histological category | 4.482 | 0.954–21.063 | 0.0574 |
| Tumour location | 0.967 | 0.087–10.722 | 0.9784 |
| Treatment group | 0.266 | 0.088–0.800 | 0.0184 |

RR: relative risk, 95% CI: 95% confidence interval.

and six from Amakusa Medical Center). Eleven and four patients were treated by radiotherapy combined with HBO at the above two institutions since 1991 and 1993 respectively. In July 1997, six patients were still alive, with a median follow-up period of 37 months. The clinical characteristics of the treated patients are summarized in Table 1. All patients were followed until death or the completion of the study and were evaluated for response, toxicity and survival. There were no differences between the two treated groups in terms of patient characteristics and treatment parameters. Biopsy surgery was performed more often in the HBO-treated patients, but this was not statistically significant. Twenty patients had a follow-up period of at least 9 months after the completion of radiotherapy (range 2–76 months).

### Response to therapy (Table 2)

Fifteen of the 29 patients responded during the follow-up periods. Only three of these had a protracted or delayed response in which continued to decrease in size for the ensuing 3–6 months. In the non-HBO group, response to radiotherapy was seen in four of 14 patients (29%). All four responders showed partial regression at the completion of the therapy, and one showed complete disappearance of the tumour 4 months later. In the HBO group, 11 of 15 patients (73%) showed a partial or complete response to the combined therapy, and nine of these showed a partial response at the completion of the therapy. The other two responders did not show more than 50% tumour regression at the completion of the therapy, but they had a protracted or delayed response 6 months later. Three responders showed complete disappearance of the residual tumour 3–6 months later. All responders received irradiation within 15 min after HBO exposure. Patients irradiated 30 min after decompression had no tumour regression, but three of their tumours remained stable for 11–14 months. The response rates were significantly different between the two treated groups ($P$ < 0.05).

### Survival analysis

In the non-HBO group, all four responders showed tumour recurrence. All 14 patients developed tumour growth or recurrence and died within 36 months after diagnosis. In the HBO group, tumour recurrence was seen in four of nine responders (two patients died from causes unrelated to the tumour). Patient survival from diagnosis ranged from 4 to 78 months, with a median of 24 months. At the time of this report, six patients were alive and three of them were last evaluated at 78, 67 and 66 months, with the first two patients having no evidence of recurrence. The histological type of all these long-term survivors was anaplastic astrocytoma. The median survival time for the HBO-treated group was 24 months versus 12 months for the non-HBO treated group (mean follow-up

© Cancer Research Campaign 1999

was 26 and 16 months respectively). The differences between survival curves and median survival time were significant ($P < 0.05$) (Figure 1).

In a Cox model analysis to investigate the relationship between prognostic factors and survival, univariate analysis showed that histological grade and age significantly increased relative risks (RR = 5.58, 95% CI: 1.584–19.660; RR = 1.05, 95% CI: 1.004–1.089 respectively), but the combined treatment significantly increased survival rate as compared with treatment without HBO (RR = 0.35, 95% CI: 0.137–0.883) (Table 3). When these prognostic factors, including tumour location, were entered into a multivariate analysis, the treatment group was the only significant prognostic factor (RR = 0.27, 95% CI: 0.088–0.800) (Table 4).

### Toxicity

As a side-effect of the chemotherapy, mild bone marrow suppression was noted in some patients, but it was not different in the two groups. Moreover, radiation necrosis and convulsive seizures were not seen in patients who received HBO. There were no other side-effects regarding HBO exposure.

### DISCUSSION

Our current pilot study shows that radiotherapy after HBO exposure improved the survival of patients with malignant gliomas in spite of the small number of patients and non-randomized investigation. The data presented here indicate that the existence of hypoxic tumour cells is important for radiotherapy of malignant gliomas and that HBO acted as a radiosensitizer.

### Tumour hypoxia

Tumour hypoxia in human malignant glioma has been studied by direct and indirect measurements. In direct $P\text{O}_2$ measurement with a tube-type Clarke electrode, Kayama et al (1991) found that the intratumoural $P\text{O}_2$ value was significantly lower than that of the surrounding brain. They also noted that there was no correlation between intratumoural $P\text{O}_2$ and tumour vascularity as demonstrated on angiograms or CT scans. More recently, Rampling et al (1994), using Eppendorf polarographic $O_2$ electrode, revealed a median $P\text{O}_2$ value of 7.4 mm Hg in ten patients with glioblastoma and the presence of a large amount of severely hypoxic cells ≤ 2.5 mmHg (9.5–68.5%). In contrast to these direct measurements, identification of tumour hypoxia is very interesting in indirect non-invasive methods using nuclear medical assays. However, a definite tendency has not yet been established using these methods. Groshar et al (1993) reported the results of single photon emission computed tomography (SPECT) with [$^{123}$I]iodoazomycin arabinoside in various histological tumours, but no evidence for hypoxia was obtained in glioblastoma. However, Valk et al (1992) showed increased uptake in two of three patients with malignant gliomas using positron emission tomography (PET) imaging of [$^{18}$F]fluoromisonidazole. These different results in SPECT and PET studies may be due to the differences of imaging techniques and tracers (Rasey et al, 1996; Parliament et al, 1997).

Tissue hypoxia generally occurs when the blood supply is inadequate to meet the demand. Because oxygen consumption is lower in malignant gliomas than in the normal brain (Ito et al, 1982; Tyler et al, 1987; Mineura et al, 1994), tumour hypoxia is induced by low tumour blood flow. Radioresistance caused by hypoxia is thought to develop by two major mechanisms: diffusion-limited or chronic hypoxia, due to reduced oxygen diffusion to regions distant from the tumour vessels (Thomlinson and Gray, 1955), and acute hypoxia caused by transient occlusion of vessels (Chaplin et al, 1986). In a recent study, Helmlinger et al (1997) measured $P\text{O}_2$ using a phosphorescence quenching microscopy technique in human tumour xenografts, and reported that hypoxic (≤ 5 mmHg) and near anoxic values (0–0.5 mmHg) were reached 70–80 and ≥ 150 μm away from tumour vessels respectively. However, a few histological studies on malignant gliomas have failed to obtain a certain tendency of tumour vascular density (Yoshii and Sugiyama, 1988; Stewart et al, 1991; Wesseling et al, 1994), although blood flow is lower in malignant gliomas than in normal white matter (Ito et al, 1982; Tyler et al, 1987; Mineura et al, 1994). These conflicting observations support the hypothesis that tumour tissue perfusion is incomplete due to transient (acute hypoxia) or partial occlusion of vessels (Chaplin et al, 1986). In fact, tumour vessels of human glioma xenografts are not fully open and the perfusion fraction of the vessels is relatively low, ranging from 20 to 85% (mean: about 60%) (Bernsen et al, 1995).

Radiosensitivity of the tumour is well known to be determined by the level of $P\text{O}_2$, and to increase markedly by means of a very small amount of oxygen (Gray et al, 1953; Hall, 1994). In the present study, different tumour responses and survival rates between the treated groups suggest that $P\text{O}_2$ levels in malignant gliomas remain elevated for a certain period after HBO exposure.

### $\text{Po}_2$ after HBO exposure

The changes of $P\text{O}_2$ which respond to HBO exposure appear to be entirely different between normal tissues and blood. Wells et al (1977), using a mass spectrometer probe which quantified the duration and magnitude of the HBO effect, found that tissue $P\text{O}_2$ changed slowly during and after $O_2$ inhalation at 2 atmospheres absolute and that the decline in $P\text{O}_2$ was slower in subcutaneous tissue than in muscle. Moreover, they noted that the subcutaneous tissue $P\text{O}_2$ value was as much as 30% greater than the control 30 min after decompression. They concluded that the different $P\text{O}_2$ changes in tissues were partially due to differences in tissue perfusion.

Many investigators have reported that metabolism in malignant gliomas is anaerobic and that the oxygen consumption rate is lower in the tumour than in normal white matter (Ito et al, 1982; Tyler et al, 1987; Mineura et al, 1994). Normal brain $P\text{O}_2$ has been noted to decrease quickly after HBO exposure in animal experiments (Jamieson and van den Brenk, 1963). $P\text{O}_2$ in malignant gliomas declines more slowly after decompression due to low oxygen consumption in addition to low blood flow. We postulated that only tumour $P\text{O}_2$ remains elevated for a substantial period after decompression. According to this hypothesis, radiotherapy after HBO exposure may increase the sensitivity of hypoxic tumour cells without an increase in normal brain injury. From our small series, this new combined treatment appears to be very effective for patients with malignant gliomas (Kohshi et al, 1996). However, the present result that no cases irradiated 30 min after decompression had tumour regression, suggests that the tumour $P\text{O}_2$ may decrease in the period between 15 and 30 min after HBO exposure to lower levels without enhancing the effect of radiotherapy. The tumour $P\text{O}_2$ change after HBO exposure is a very important subject for future study.

© Cancer Research Campaign 1999

### Radiation during HBO exposure

Many clinical trials have been performed in cancer treatments of radiotherapy combined with HBO. The previous combined method was a repetitive administration of irradiation during HBO exposure. In some clinical trials, significant improvements in local control and survival have been seen in cancers of head and neck, cervical region and uterine cervix (Henk et al, 1977; Watson et al, 1978; Henk, 1986), but no benefit has been demonstrated in patients with cancers of skin, bronchus and bladder (Sealy et al, 1974; Cade and McEwen, 1978). There are only two reports of human malignant gliomas treated with this therapeutic method (Chang, 1977; Dowling et al, 1992). One of them showed that the median survival rate at 18 months appeared considerably higher in the HBO group (28%) than in the control (10%) (Chang, 1977). Two of 38 patients who received HBO lived more than 4 years. One patient who died from radiation necrosis of the brain was identified as having no microscopic evidence of residual tumour. Irradiation during HBO exposure had been recognized as a promising therapeutic method for treating malignant gliomas. For this regimen, however, irradiating in a pressure chamber required sedation before each irradiation and myringotomies (Chang, 1977). The irradiated region was the whole brain and it was difficult to reduce the local area to the tumour field. Unfortunately, serious side-effects, such as radiation necrosis and convulsive seizures, were induced in some patients (Chang, 1977; Jain, 1990). Therefore, this regimen has not been used as a standard treatment for malignant gliomas (Jain, 1990; Dische, 1991).

### Radiation after HBO exposure

Our new therapeutic regimen, irradiating after HBO exposure, is very simple and safe for the patients so myringotomy and sedation are not required. The combination with HBO produced a significant reduction of residual tumour, and a response was seen in all patients irradiated immediately after decompression. In studies assessed radiographically, patients who responded to radiotherapy tended to live longer than non-responders (Murovic et al, 1986; Wood et al, 1988; Barker et al, 1996). Although our series represents non-randomized data in a small number of patients, the treated groups did not differ substantially. Nevertheless, we significantly prolonged the survival time in the HBO group. Prognostic factors in malignant gliomas are generally well known to be histology, age, KPS, prior surgery, mental status and time from first symptom (Burger and Green, 1987; Curran et al, 1993). Multivariate analysis in our small series revealed that combination with HBO was a good predictive prognostic factor for survival. Radiotherapy after HBO exposure is one of the initial treatment options for patients with malignant gliomas. However, all four patients irradiated 30 min after decompression showed no tumour regression and died due to tumour growth. The timing of irradiation may be very important in this regimen and irradiation should be done immediately after HBO exposure in order to improve the therapeutic effects.

The results of this trial suggest that HBO can increase the efficacy of radiotherapy in malignant gliomas. In order to provide more convincing evidence of this, randomized clinical trials must be undertaken.

### ACKNOWLEDGEMENTS

This work was supported by funds from the University of Occupational and Environmental Health and Daido Hoxan Inc, and Grant-in-Aid for Scientific Research 10877221 from the Ministry of Education, Science, Sports and Culture, Japan.

### REFERENCES

Barker FG, Prados MD, Chang SM, Gutin PH, Lamborn KR, Larson DA, Malec MK, McDermott MW, Sneed PK, Wara WM and Wilson CB (1996) Radiation response and survival time in patients with glioblastoma multiforme. *J Neurosurg* **84**: 442–448

Bernsen HJJA, Rijken PFJW, Oostendorp T and van der Kogel AJ (1995) Vascularity and perfusion of human gliomas xenografted in the athymic nude mouse. *Br J Cancer* **71**: 721–726

Burger PC and Green SB (1987) Patient age, histologic features, and length of survival in patients with glioblastoma multiforme. *Cancer* **59**: 1617–1625

Cade IS and McEwen JB (1978) Clinical trials of radiotherapy in hyperbaric oxygen at Portsmouth, 1964–1976. *Clin Radiol* **29**: 333–338

Chang CH (1977) Hyperbaric oxygen and radiation therapy in the management of glioblastoma. *Natl Cancer Inst Monogr* **46**: 163–169

Chaplin DJ, Durand RE and Olive PL (1986) Acute hypoxia in tumors: implications for modifiers of radiation effects. *Int J Radiat Oncol Biol Phys* **12**: 1279–1282

Cox DR (1972) Regression models and life tables. *J R Stat Soc* **34**: 187–220

Curran WJ, Scott CB, Horton J, Nelson JS, Weinstein AS, Fischbach AJ, Chang CH, Rotman M, Asbell SO, Krisch RE and Nelson DF (1993) Recursive partitioning analysis of prognostic factors in three radiation therapy oncology group malignant glioma trials. *J Natl Cancer Inst* **85**: 704–710

Dische S (1991) What have we learnt from hyperbaric oxygen? *Radiother Oncol* **20**: 71–74

Dowling S, Fischer JJ and Rockwell S (1992) Fluosol and hyperbaric oxygen as an adjunct to radiation therapy in the treatment of malignant gliomas: a pilot study. *Biomater Artif Cells Immobilization Biotechnol* **20**: 903–905

Fowler JF (1989) The linear-quadratic formula and progress in fractionated radiotherapy. *Br J Radiol* **62**: 679–694

Gray LH, Conger AD, Ebert M, Hornsey A and Scott OCA (1953) The concentration of oxygen dissolved in tissues at the time of irradiation as a factor in radiotherapy. *Br J Radiol* **26**: 638–648

Groshar D, McEwan AJB, Parliament MB, Urtasun RC, Golberg LE, Hoskinson M, Mercer JR, Mannan RH, Wiebe LI and Chapman JD (1993) Imaging tumor hypoxia and tumor perfusion. *J Nucl Med* **34**: 885–888

Hall EJ (1994) *Radiobiology for the Radiologist*, pp. 133–152. Lippincott: Philadelphia

Helmlinger G, Yuan F, Dellian M and Jain RK (1997) Interstitial pH and $pO_2$ gradients in solid tumors in vivo: high-resolution measurements reveal a lack of correlation. *Nature Med* **3**: 177–182

Henk JM (1986) Late results of a trial of hyperbaric oxygen and radiotherapy in head and neck cancer: a rationale for hypoxic cell sensitizers? *Int J Radiat Oncol Biol Phys* **12**: 1339–1341

Henk JM, Kunkler PB and Smith CW (1977) Radiotherapy and hyperbaric oxygen in head and neck cancer. Final report of first controlled clinical trial. *Lancet* **2**: 101–103

Ito M, Lammertsma AA, Wise RJS, Bernardi S, Frackowiak R, Hckenzie C, Thomas DGT and Jones T (1982) Measurement of regional cerebral blood flow and oxygen utilization in patients with cerebral tumours using $^{15}O$ and positron emission tomography: analytical techniques and preliminary results. *Neuroradiology* **23**: 63–74

Jain KK (1990) *Textbook of Hyperbaric Medicine*, pp. 408–417. Hogrefe & Huber Publishers: Toronto

Jamieson D and van den Brenk HAS (1963) Measurement of oxygen tensions in cerebral tissues of rats exposed to high pressures of oxygen. *J Appl Physiol* **18**: 869–876

Kaplan EL and Meier P (1958) Non-parametric estimation from incomplete observations. *J Am Stat Assoc* **53**: 457–481

Karnofsky DA, Abelmann WH and Craver LS (1948) The use of the nitrogen mustards in the palliative treatment of carcinoma: with particular reference to bronchogenic carcinoma. *Cancer* **1**: 634–656

Kayama T, Yoshimoto T, Fujimoto S and Sakurai Y (1991) Intratumoral oxygen pressure in malignant brain tumor. *J Neurosurg* **74**: 55–59

Kleihues P, Burger PC and Scheithauer BW (1993) *Histological Typing of Tumours of the Central Nervous System*, 2nd Ed. Springer-Verlag: Berlin

Kohshi K, Kinoshita Y, Terashima H, Konda N, Yokota A and Soejima T (1996) Radiotherapy after hyperbaric oxygenation for malignant gliomas: a pilot study. *J Cancer Res Clin Oncol* **122**: 676–678

 © Cancer Research Campaign 1999

Mantel N (1966) Evaluation of survival data and two new rank order statistics arising in its consideration. *Cancer Chemother Rep* **50**: 163–170

Mineura K, Sasajima T, Kowada M, Ogawa T, Hatazawa J, Shishido F and Uemura K (1994) Perfusion and metabolism in predicting the survival of patients with cerebral gliomas. *Cancer* **73**: 2386–2394

Murovic J, Turowski K, Wilson CB, Hoshino T and Levin V (1986) Computerized tomography in the prognosis of malignant cerebral gliomas. *J Neurosurg* **65**: 799–806

Parliament MB, Franko AJ, Allalunis-Turner MJ, Mielke BW, Santos CL, Wolokoff BG and Mercer JR (1997) Anomalous patterns of nitroimidazole binding adjacent to necrosis in human glioma xenografts: possible role of decreased oxygen consumption. *Br J Cancer* **75**: 311–318

Rampling R, Cruickshank G, Lewis AD, Fitzsimmons SA and Workman P (1994) Direct measurement of $pO_2$ distribution and bioreductive enzymes in human malignant brain tumors. *Int J Radiat Oncol Biol Phys* **29**: 427–431

Rasey JS, Koh W-J, Evans ML, Peterson LM, Lewellen TK, Graham MM and Krohn KA (1996) Quantifying regional hypoxia in human tumors with positron emission tomography of [$^{18}$F]fluoromisonidazole: a pretherapy study of 37 patients. *Int J Radiat Oncol Biol Phys* **36**: 417–428

Sealy A, Hockly J and Shepstone B (1974) The treatment of malignant melanoma with cobalt and hyperbaric oxygen. *Clin Radiol* **25**: 211–215

Stewart PA, Farrell CL and Del Maestro RF (1991) The effect of cellular microenvironment on vessels in the brain. Part 1: vessel structure in tumour, peritumour and brain from humans with malignant glioma. *Int J Radiat Biol* **60**: 125–130

Thomlinson RH and Gray LH (1955) The histological structure of some human lung cancers and the possible implications for radiotherapy. *Br J Cancer* **9**: 539–549

Tyler JL, Diksic M, Villemure J-G, Evans AC, Meyer E, Yamamoto YL and Feindel W (1987) Metabolic and hemodynamic evaluation of gliomas using positron emission tomography. *J Nucl Med* **28**: 1123–1133

Valk PE, Mathis CA, Prados MD, Gilbert JC and Budinger TF (1992) Hypoxia in human gliomas: demonstration by PET with fluorine-18 fluoromisonidazole. *J Nucl Med* **33**: 2133–2137

Watson ER, Halnan KE, Dische S, Saunders MI, Cade IS, McEwen JB, Wiernik G, Perrins DJ and Sutherland I (1978) Hyperbaric oxygen and radiotherapy: a Medical Research Council trial in carcinoma of the cervix. *Br J Radiol* **51**: 879–887

Wells CH, Goodpasture JE, Horrigan DJ and Hart GB (1977) Tissue gas measurements during hyperbaric oxygen exposure. In: *Procceedings of the 6th International Congress on Hyperbaric Medicine*, Smith G (ed), pp. 118–124. Aberdeen University Press: Aberdeen

Wesseling P, van der Laak JAWM, De Leeuw H, Ruiter DJ and Burger PC (1994) Quantitative immunohistological analysis of the microvasculature in untreated human glioblastoma multiforme. *J Neurosurg* **81**: 902–909

Wood JR, Green SB and Shapiro WR (1988) The prognostic importance of tumor size in malignant gliomas: a computed tomographic scan study by the Brain Tumor Cooperative Group. *J Clin Oncol* **6**: 338–343

Yoshii Y and Sugiyama K (1988) Intercapillary distance in the proliferating area of human glioma. *Cancer Res* **48**: 2938–2941

© Cancer Research Campaign 1999
